# Human Papilloma Virus Identification in Breast Cancer Patients with Previous Cervical Neoplasia

**DOI:** 10.3389/fonc.2015.00298

**Published:** 2016-01-08

**Authors:** James S. Lawson, Wendy K. Glenn, Daria Salyakina, Rosemary Clay, Warick Delprado, Bharathi Cheerala, Dinh D. Tran, Christopher C. Ngan, Shingo Miyauchi, Martha Karim, Annika Antonsson, Noel J. Whitaker

**Affiliations:** ^1^School of Biotechnology and Biomolecular Science, University of New South Wales, Sydney, NSW, Australia; ^2^Center for Computational Science, University of Miami, Miami, FL, USA; ^3^Douglass Hanly Moir – Pathology, Macquarie Park, NSW, Australia; ^4^QIMR Berghofer Medical Research Institute, Brisbane, QLD, Australia

**Keywords:** breast cancer, cervical neoplasia, human papilloma viruses, young age

## Abstract

**Purpose:**

Women with human papilloma virus (HPV)-associated cervical neoplasia have a higher risk of developing breast cancer than the general female population. The purpose of this study was to (i) identify high-risk HPVs in cervical neoplasia and subsequent HPV positive breast cancers which developed in the same patients and (ii) determine if these HPVs were biologically active.

**Methods:**

A range of polymerase chain reaction and immunohistochemical techniques were used to conduct a retrospective cohort study of cervical precancers and subsequent breast cancers in the same patients.

**Results:**

The same high-risk HPV types were identified in both the cervical and breast specimens in 13 (46%) of 28 patients. HPV type 18 was the most prevalent. HPVs appeared to be biologically active as demonstrated by the expression of HPV E7 proteins and the presence of HPV-associated koilocytes. The average age of these patients diagnosed with breast cancer following prior cervical precancer was 51 years, as compared to 60 years for all women with breast cancer (*p* for difference = 0.001).

**Conclusion:**

These findings indicate that high-risk HPVs can be associated with cervical neoplasia and subsequent young age breast cancer. However, these associations are unusual and are a very small proportion of breast cancers. These outcomes confirm and extend the observations of two similar previous studies and offer one explanation for the increased prevalence of serious invasive breast cancer among young women.

## Introduction

Women with squamous or glandular precancer of the cervix have a significantly higher risk of subsequent breast cancer than the general female population – odds ratios 1.10 and 1.52, respectively (1). Women with human papilloma virus (HPV)-associated cervical neoplasia may some years later develop HPV-associated breast cancer (2, 3).

Although these data indicate that HPVs may have a possible role in some breast cancers, it is unlikely that HPVs have a major causal role. There are two reasons for this view: (i) in immunocompromised patients (due to either human immunodeficiency viral infections or post organ transplantation therapy), there is no increased prevalence of breast cancer, which is in contrast to the twofold to sixfold increased prevalence of HPV-associated cervical cancer in these patients (4), and (ii) the HPV viral load in breast cancer is extremely low (5, 6).

Data from The Cancer Genome Atlas (TCGA), published on line by the Larsson Lab (6), indicate a low prevalence of high cancer risk HPVs in breast cancer. It has been shown that approximately 1.3% of these data are contaminated by HPV 18 (7, Cantalupo, 2015, personal communication).

There is an apparent conflict between (i) data which indicate an increased risk of breast cancer among women with prior HPV-associated cervical pathology (1–3) and (ii) the very low prevalence of high-risk HPVs in breast cancer as shown in the analyses of the TCGA breast cancer data (6). Therefore, there is value in confirming by modern molecular methods that the two previous studies which demonstrated that some women with HPV-associated cervical cancer could subsequently develop same type HPV positive breast cancer (2, 3) and in determining if, despite their extremely low concentration, HPVs in breast cancer are biologically active and have potential oncogenic influences. HPV biological activity can be assessed in three ways (i) by the identification of HPV transcripts (RNA transcripts have been identified in the TCGA analyses referred to above), (ii) expression of the HPV oncogenic protein HPV E7 in breast cancers, and (iii) the identification of HPV-associated koilocytes in breast cancer.

The purpose of this study was to (i) identify high-risk HPVs in cervical neoplasia and subsequent HPV positive breast cancers which developed in the same patients and (ii) determine if these HPVs were biologically active.

## Research Approaches, Materials and Methods

### Research Approaches

We used the following research approaches: (i) a retrospective cohort study of women with breast cancer who had prior precancer or neoplasia of the cervix, (ii) a study of HPV-associated biomarkers in these breast cancers to determine if the HPVs were biologically active, and (iii) identification of koilocytes by light microscopy. We also compared the outcomes of these studies with normal breast tissues from women who had not developed breast cancer after approximately 10 years.

### Patients and Specimens

All cervical and subsequent breast cancer specimens were from women residing in Australia. We reviewed approximately 4,000 pathology reports of breast cancer and identified 28 patients who had either HPV cervical infections or more commonly cervical intraepithelial neoplasia (CIN) 1–11 years prior to developing breast cancer. Archival formalin-fixed specimens from these patients were identified and collected from a large Australian pathology service (Douglass Hanly Moir – Pathology).

Eighteen unselected archival formalin-fixed “normal” breast specimens from women who had breast reduction surgery were used as a comparison group. Normal breast specimens from these women do not provide a strict control group as there is no information available about their cervical tissues, although cervical HPV infections are likely to be present in some of these women (8). While it is possible “normal” breast tissues from breast reduction or enhancement are not necessarily normal, they are not malignant (9). These donors were followed for 10 or more years via the New South Wales State Cancer Registry and none had developed breast cancer.

#### Cohort Studies

We sought to identify HPV DNA sequences in prior cervical neoplasia and later breast cancer specimens from the same patients to determine if the same HPV virus was present. If the HPV DNA was of the same type and sequences in both the prior cervical as in the later breast cancer tissues from the same subject, this provides potential evidence of prior HPV infection as a possible cause for malignancy in the breast.

### Preparation of Genomic DNA

For standard and quantitative polymerase chain reaction (PCR), genomic DNA (gDNA) was extracted from formalin-fixed paraffin-embedded (FFPE) breast and cervix tissues using a combination of heat treatment and a DNA extraction kit (DNeasy Blood and tissue extraction, QIAGEN). The method is described in Steinau et al. (10). DNA extraction was conducted according to the manufacturer’s recommendation.

A blank extraction control (an extraction without FFPE tissues) was performed during the DNA extraction procedure. The gDNA extracts were quantified with a NanoDrop spectrophotometer (Thermo Scientific). The quality of gDNA was assessed by amplification of human β-actin. The primers used were β-actin5′_fwd (5′-CTTCTGCCGTTTTCCGTAGG-3′) and β-actin3′_rev (5′-TGGGATGGGGAGTCTGTTCA-3′) at the final concentration of 1 μM. Thermal cycles were 94°C for 15 min; 94°C for 30 s, 55°C for 30 s, and 72°C for 45 s for 30 cycles. HotStarTaq Master Mix Kit (QIAGEN) was used for PCR master mix. gDNA samples, which were β-actin positive, were selected for the detection of HPV genomes.

#### Polymerase Chain Reaction

Both semi-nested PCR and real-time quantitative PCR were used for the detection of HPV. Archival DNA is fragmented and the original nested MY/Gp primers, in the L1 gene, did not always give sound results due to the length of the MY product. Therefore, a semi-nested PCR, MY11 to Gp6, followed by Gp5–Gp6 was used. Some of the primers have been altered slightly and are degenerate for HPV16 and 18. They are also capable of bringing up types 3, 11, 12, 45, 58, 73, and 75.

MY11 – (5′GCACAGGGYCAYAAYAATGG3′)
Gp5 – (5′TATTTGTTACTGTKGTWGATAC3′)
Gp6 – (5′GACATGKKGAGGAATATGATT3′)

Positive PCR products were routinely sequenced in addition to the negative controls, to ensure there was no contamination. The negative controls were no DNA (water) and a blank extraction (which tested the reagents), plus sequencing of the products of these controls in case the bands could not be seen on a gel. Negative outcomes of PCR analyses of selected breast cancer specimens provided negative controls based on breast tissues.

### Real-Time PCR

The HPV L1 gene in gDNA samples was amplified using a real-time PCR machine (Rotor Gene Q, QIAGEN). Thermal cycles used were 95°C for 5 min, 95°C for 10 s, and 60°C for 30 s for 60 cycles. The PCR reaction mix was the Quanti Fast SYBR Green kit (QIAGEN) and was used according to the manufacturer’s recommendation. The primers used for detection of HPV were *GP05*+ *forward* (5′TTTGTTACTGTGGTAGATACTAC3′); and *GP06*+ *reverse* (5′AAATCATATTCCTCMMCATGTC3′) at the final concentration of 1 μM.

The quantity of gDNA used for PCR was determined by estimating theoretical gene copies of HPV 16 in breast and breast cancer tissues (5). A positive control for HPV was a purified PCR product containing the HPV L1 region. It was amplified with MY11 and MY09 primers. Negative controls were (i) ultrapure H_2_O (*no DNA template*) and (ii) extraction blank (a DNA extraction control without FFPE tissues).

Polymerase chain reaction products were analyzed and selected for sequencing based on amplification profiles and the achievement of the known melting point (the specific temperature for each product). The analysis was performed using Rotor Gene Q software (QIAGEN).

### Independent Confirmation of the PCR-Based Outcomes

DNA extracts from 11 selected (for positive HPV) cervical precancer and breast cancer specimens were independently analyzed for the presence of HPV DNA (Annika Antonnson, QIMR Berghofer Medical Research Institute), using methods as described in Ref. (11). Different methods to those described above were used for these confirmatory analyses (11).

### Sequencing the PCR Products and Identification of HPV Types

The HPV PCR products from GP5 to Gp6 were sequenced to determine the HPV type. The HPV genotypes were identified by BLAST via the US National Center for Biotechnology Information.

### *In situ* PCR

Archival tissues on slides were washed in xylene to remove the wax followed by washes in decreasing concentrations of alcohol. The tissues were subjected to pepsin digestion with varying times of digestion which were required for different tissues. These differences were probably due to fixation procedures, which could vary in duration. The digestion was stopped in 0.1M Tris buffer pH 8. Approximately 75 μl of PCR mix, containing inner nested PCR primers Gp5–Gp6. Digoxogenin (DIG) – dUTP (0.3 nM) (Roche) – was added to the tissue in a frame which was sealed. PCR cycling was the same as for standard PCR. Detection using Anti-DIG AP-Fab fragments (1 μl) (Roche) in buffer pH 7.5 followed by NBT/BCIP (2 μl) (Roche) in buffer pH 9.5 was stopped when a blue color was observed in the cells of the cancer specimen and not in the negative control. The tissues were counterstained with eosin. We used stringent negative controls that included omitting both DNA primers and Taq polymerase when conducting *in situ* PCR. Any specimens that were positive in the negative controls showed that the DNA was self-priming and were unsuitable for *in situ* work (this is probably due to fragmented DNA acting as primers). These samples were eliminated from the study. Negative specimens were subjected to beta-globin *in situ* PCR to confirm the result.

#### Identification of HPVs

The identification of HPV in normal and benign breast or breast cancer specimens was considered as positive if two or more of the following outcomes in the same specimen were observed: (i) HPV DNA sequences identified by standard PCR and/or real-time PCR and (ii) HPV positive *in situ* PCR.

#### HPV Biological Activity

An assessment of HPV biological activity can be conducted in several ways: (i) the identification of HPV oncoprotein E7 and (ii) the identification of HPV-associated koilocytes.

### Immunohistochemistry

Antibodies specific for HPV E7 proteins, have recently been developed and were used in this study. These antibodies were HPV E7 monoclonal “Cervimax” – Valdospan GmbH. Austria (12, 13). Standard manual immunohistochemistry (IHC) methods were used to identify HPV E7 proteins, with the omission of the antigen retrieval step. The specificity of these antibodies has been demonstrated experimentally and by epidemiological studies (12, 13). The HPV E7 antibody reacts with a wide range of HPV types, including high risk for cancer HPV 16 and 18. HPV E7 antibodies have not been previously used on breast tissues. Good outcomes for both HPV E7 were achieved with clear staining of both cytoplasm and nuclei of breast cancer cells with 1:100, or 1:50 dilution of the antibody without antigen retrieval (antigen retrieval involves heating of the specimens at 95°C). Positive controls for the E7 antibody were the HeLa (HPV18) cell line and cervical tissues that were positive for HPV sequences by PCR and sequencing. Freshly cut slides and the cell lines needed lesser antibody (1/500 for 30 min) than recommended by the manufacturer (Valdospan). Slides that were up to 5 years old needed 1/100 dilution of antibody for 1 h.

#### Histology

The identification of koilocytes by light microscopy is subjective. For this reason, two independent observers (James S. Lawson and Warick Delprado) reviewed all the specimens and assessed the presence of koilocytes.

### Statistics

The SPSS statistical package was used to (i) assess the significance of any differences in outcomes for the various investigations and (ii) assess for correlation of non-parametric data.

## Results

### HPV Identification in Cervical and Breast Pathology in the Same Australian Patients

**Figure 1:**
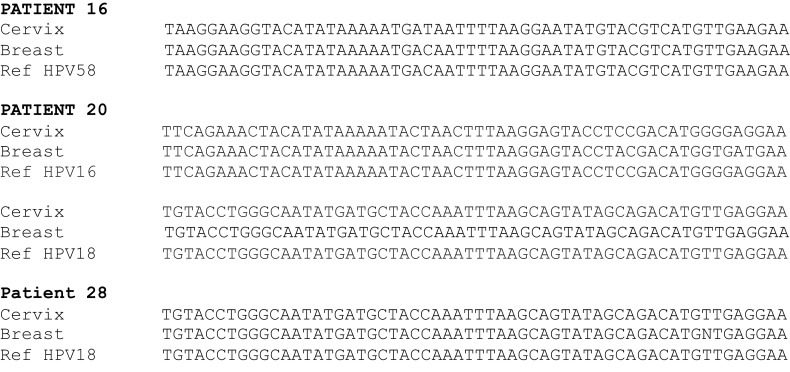
**Examples of 3′ PCR HPV DNA sequences in cervical intraepithelial neoplasia and in subsequent breast cancer in three patients**. Patient 20 was a mixture of HPV 16 and 18 extracted from different blocks. The reference sequences are HPV 16 FJ610152., HPV 18 Q902111, and HPV 58 HM639516. The total length of sequence (approximately 100 bases) did not show any differences to the reference sequences.

**Figure 2:**
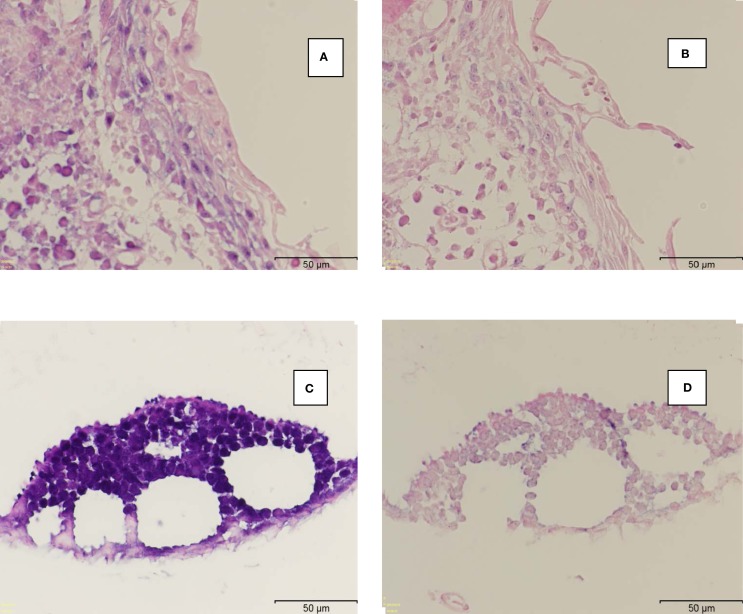
**Human papilloma virus positive cervical intraepithelial neoplasia followed by breast cancer in the same patient**. HPV identified by *in situ* PCR in cervical intraepithelial neoplasia grade 1 (CIN 1) diagnosed in a patient who later developed HPV positive ductal carcinoma *in situ* breast cancer (DCIS). Both were confirmed to be HPV 18 by PCR followed by sequencing. **(A)** HPV positive CIN 1. **(B)** Negative control with PCR primer omitted. **(C)** HPV positive ductal carcinoma *in situ*. **(D)** Negative control with PCR primer omitted.

The same high-risk HPV types were identified in both the cervical and breast specimens from 13 (46%) of patients. High-risk HPV types 16, 18, 33, and 58 were identified in 19 (70%) invasive and 2 (100%) non-invasive breast cancer specimens (Table 1). HPV type 18 was the most prevalent. This compares with the prevalence of HPV sequences in 3 (17%) of 18 normal breast specimens from cosmetic surgery (Table 2) (*p*-value for difference = 0.001). The HPV 18 DNA sequences in both cervical neoplasia and later breast cancer are shown in Figure 1. These data demonstrate that (i) the sequences are virtually identical in both the cervical and subsequent breast cancers from the same patients and offers direct evidence that prior HPV cervical infection is the likely source of the later HPV positive breast cancer and (ii) there are variations in the sequences, which indicate the outcomes are valid and not a result of contamination. This is illustrated in Figure 2 showing HPV, positive by *in situ* PCR, in which HPV type 18 was identified in cervical intraepithelial neoplasia grade 1 (CIN 1) diagnosed in a patient who later developed HPV type 18 positive ductal carcinoma *in situ* breast cancer (DCIS).

**Table 1:** **HPV identification in cervical neoplasia and subsequent breast cancer**.

Patient	Age	Diagnosis	HPV E7	HPV

			Immunohistochemistry	PCR consolidated
1. Cervix	46	CIN1		HPV 16/18
Breast	48	IDC	Neg	HPV 18

2. Cervix	60	Normal		Neg
Breast	66	IDC Gr3	Neg	HPV 18

3. Cervix	62	CIN1		HPV 18
Breast	73	DCIS	2+	HPV 18

4. Cervix	53	CIN3		HPV 18
Breast	62	IDC Gr2		

5. Cervix	44	CIN2		HPV 18
Breast	45	IDC Gr2	Neg	HPV 18

6. Cervix	42	HPV koilos		Neg
Breast	47	IDC Gr2	3+	HPV 18

7. Cervix	39	CIN3		
Breast	45	ILC Gr2	3+	Neg

8. Cervix	40	CIN3		HPV 18
Breast	45	IDC Gr3	3+	HPV 18

9. Cervix	33	CIN2		HPV 18
Breast	39	IDC Gr2	3+	HPV 18

10. Cervix	44	HPV koilos		HPV 18
Breast	44	DCIS	Neg	HPV 18

11. Cervix	57	HPV koilos		HPV 18
Breast	68	IDC Gr3	Neg	HPV 18

12. Cervix	43	CIN3		HPV 16/18
Breast	45	IDC Gr2		HPV 18

13. Cervix	29	CIN 2		HPV 16
Breast	32	IDC	3+	HPV 18

14. Cervix	46	HPV koilos		HPV 18
Breast	47	IDC Gr2	1+	HPV 18

15. Cervix	41	CIN1		
Breast	46	IDC Gr1	2+	HPV 18

16. Cervix	41	CIN2		HPV 16/18/58
Breast	50	IDC Gr2	1+	HPV 58

17. Cervix	33	CIN3		HPV 18
Breast	34	IDC Gr3	Neg	HPV 18

18. Cervix	29	HPV koilos		Neg
Breast	38	I Muc C Gr2		HPV 16/18

19. Cervix	55	CIN1		
Breast	62	IDC Gr1	2+	HPV 18

20. Cervix	57	CIN1		HPV 18
Breast	67	IDC Gr1	1+	HPV 16/18

21. Cervix	36	CIN1		Neg
Breast	40	IDC Gr1	1+	Neg

22. Cervix	54	CIN1		HPV
Breast	64	IDC Gr2	2+	HPV 18

23. Cervix	39	CIN2		HPV
Breast	47	ILC Gr2	1+	Neg

24. Cervix	46	CIN3		HPV 16/18
Breast	52	IDC Gr1	Neg	HPV 18

25. Cervix	53	CIN1		HPV 18
Breast	61	IDC Gr2/DCIS	3+	Neg

26. Cervix	44	CIN3		Neg
Breast	45	IDC Gr3		Neg

27. Cervix	44	Normal		HPV 18
Breast	48	DCIS	3+	HPV 18

28. Cervix	50	HPV koilos		HPV 18
Breast	61	IDC Gr1	Neg	HPV 16/18

*PCR and immunohistochemistry results using different techniques for patients 1–28*.

*CIN, cervical intraepithelial neoplasia; DCIS, ductal carcinoma *in situ*; IDC, invasive ductal carcinoma; ILC, invasive lobular carcinoma; Gr, grade*.

*Genomic extractions were tested for PCR availability before proceeding to screen for HPV. Not all extractions were successful. A different extraction was used for the real-time PCR. Not all specimens were suitable for *in situ* PCR*.

**Table 2:** **Prevalence of high-risk HPV sequences, HPV E7 protein expression, and koilocytes in normal breast tissues, cervical precancers, and subsequent cancerous breast tissues from the same patients**.

	Normal breast	Cervical precancer	Breast DCIS	Breast IDC/ILC
High-risk HPV sequences (types 16, 18, 33, 58)	3/18 (17%)	20/25 (80%)	2/2	19/27 (70%)
HPV E7	4/18 (22%)		1/2	16/25 (64%)
Koilocytes	0/18 (0%)	27/28 (96%)	1/2	8/25 (32%)

*Breast cancer developed 1–11 years after the initial cervical condition (hyperplasia with HPV characteristics or CIN I, II, or III)*.

*DCIS, ductal carcinoma *in situ*; IDC, invasive ductal carcinoma; ILC, invasive lobular carcinoma*.

### Confirmation of These Data

DNA extracts from 11 cervical and breast cancer HPV positive specimens (as identified in the University of New South Wales laboratories) were independently analyzed (Annika Antonsson, QIMR Berghofer Medical Research Institute). HPV types 16, 18, and 33 were identified in 4 cervical and 2 breast specimens. The identification of different HPV types indicates that contamination is improbable.

### Cervical and Breast Pathology

The pathological characteristics of the prior cervical and later breast specimens from the same patient are shown in Table 1. Two cervical specimens were normal, 6 of 28 (21%) had HPV koilocytosis (no CIN); 8 of 28 (31%) were CIN grade 1; 5 of 28 (17%) were CIN 2; and 7 of 28 (21%) were CIN 3. Two of the breast cancers which developed 1–11 years later were non-invasive and 26 of 28 (93%) were invasive. Six of 28 (21%) of the invasive breast cancers were grade 1, 11 of 28 (39%) grade 2 and 6 of 28 (21%) grade 3.

### Age of Patients at Date of Diagnosis

The average age of patients at date of diagnosis of breast cancer following cervical pathology was 51 years (Table 1). This is on average 9 years younger than the average age of 60 years at diagnosis of all breast cancers in Australian women (*p*-value = 0.001). Eleven of 28 (39%) of the patients were 45 years or younger at the date of breast cancer diagnosis.

The number of years between the age of cervical and subsequent breast cancer diagnosis was 3.7 years for women below the age of 50 and 9.0 years for women 50 years or over (*p*-value = 0.001). The reason for the much lower gap for younger women in years between cervical and later breast cancer in the same patient is not known.

### Immunohistochemistry

As shown in Tables 1 and 2, both HPV sequences and HPV E7 protein expression were present in 12 of 24 (50%) of breast cancer specimens. In 8 of 24 (33%) of breast cancer specimens, HPV sequences were identified but HPV E7 protein was not expressed. HPV E7 protein was expressed in 4 of 18 (22%) normal breast specimens from cosmetic surgery (*p*-value = 0.007).

**Figure 3:**
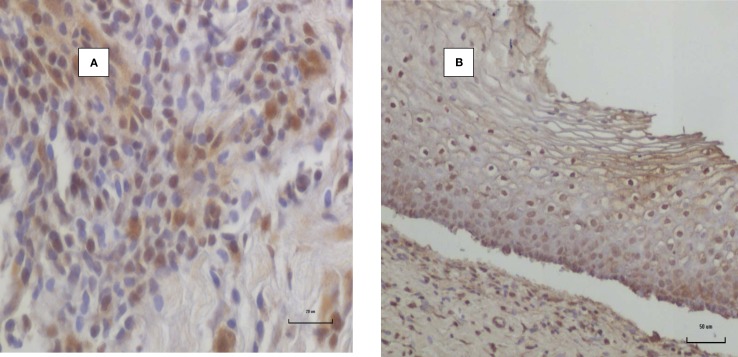
**HPV E7 protein expression invasive breast cancer**. **(A)** HPV 18 positive invasive breast cancer in a woman aged 48 years. **(B)** Positive control HPV positive cervical intraepithelial neoplasia.

HPV E7 in an HPV positive invasive breast cancer specimen is shown in Figure 3. The HPV E7 staining characteristics of HPV E7 in the CIN control specimen are virtually identical with those published by Faoro et al. (12). HPV E7 staining of both the cell nuclei and cytoplasm is common in both normal breast lobules (adjacent to invasive breast cancer) and in invasive breast cancer. We have consistently observed that with respect to IHC analyses, the staining by E7 antibodies is much stronger in normal breast tissues and benign breast tissues than in invasive cancers as is shown in Figure 3.

### HPV Biological Activity

As indicated above, HPV E7 protein was expressed in 64% of the breast cancer specimens (Table 2) and 22% of normal breast specimens from cosmetic surgery (*p*-value = 0.007).

**Figure 4:**
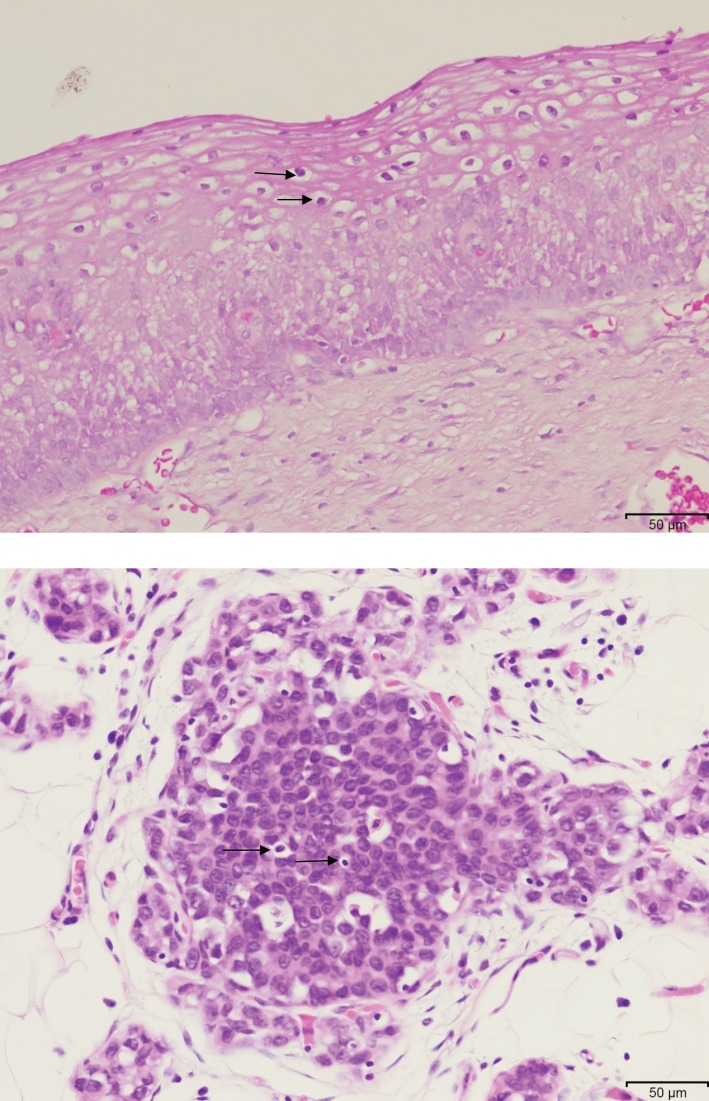
**HPV-associated koilocytes in cervical CIN 1 and putative koilocytes in ductal carcinoma *in situ* breast cancer which developed 4 years later in the same patient then aged 48 years**.

Koilocytes were identified in all of the cervical specimens and in 9 of 28 (32%) of the breast cancers, which had later developed in the same patients. Typical koilocytes are shown in squamous epithelial cells of cervical tissue and glandular epithelial cells of breast cancer in the same patient in Figure 4. As koilocytes are specific to HPV infections and are a consequence of the influence of HPV E5 and E6 genes, the presence of koilocytes in breast cancer cells is a strong indicator of HPV precancerous biological activity. The histological diagnosis of koilocytes is subjective because they are difficult to distinguish from apoptopic epithelial cells, and therefore these data need to be considered with caution.

## Discussion

Cohort studies are a valuable approach to the study of viruses and cancer because they offer insights into the natural history of the disease. Prospective cohort studies are usually preferred to case control or retrospective studies because the risk of bias is generally considered to be less. However, we have undertaken a retrospective cohort study, because it is a practical and low cost method of achieving sound outcomes.

We have shown that (i) high-risk HPVs are present in 70% of breast cancers among women who had prior HPV positive cervical neoplasia – HPV types were identical in both prior cervical neoplasia and later breast cancer in 46% of the patients, (ii) HPV E7 was expressed in 64% of invasive breast cancers, and (iii) HPV-associated koilocytes were identified in 32% of breast cancers which is an additional indicator of HPV biological activity. We have also demonstrated that HPV positive breast cancer following HPV-associated cervical cancer occurs significantly more commonly in younger age women.

Despite reviewing approximately 4,000 pathology reports of breast cancer specimens, only 28 (0.7%) women with breast cancer had prior cervical neoplasms. While this may be an under estimate because not all biopsy and surgical specimens would have been referred to the same pathology service, it suggests that prior HPV cervical neoplasia is associated with a very low prevalence of subsequent HPV positive breast cancer.

### Validity of the Findings

The use of a range of techniques, namely, standard and real-time PCR, *in situ* PCR, IHC, and koilocytosis based on light microscopy, has given broadly similar outcomes. The identification of different HPV types and the sequence variations also indicates that the outcomes by various PCR methods are valid and are not due to contamination. In addition, these observations validate the findings of the previous studies by Hennig et al. (2) and Widschwendter et al. (3), which identified high-risk HPVs in both prior cervical and later breast cancer in the same patient.

#### Interpretation of the Findings

It is particularly noteworthy that virtually identical HPV type 18 and HPV type 58 sequences were identified in both CIN and 1–11 years later in breast cancer in 46% of the same subjects. This is compatible with the same virus infection having an oncogenic role in both the cervical and breast cancers in the same patients. HPV type 18 is the dominant HPV type in both breast and prostate cancers among Australian patients (14, 15). In addition, HPV type 18 is tropic to glandular (as distinct from squamous) cervical cancer cells (16). The high (62%) proportion of HPV type 18 is very similar to the proportion of HPV type 18 identified in adenocarcinoma of the cervix (16).

The identification of koilocytes in 32% of the breast cancer specimens is of interest. This is a high proportion of koilocytes as compared to the identification of <5% in other series of breast cancers without prior HPV-associated cervical neoplasia (17). This may be due to a higher HPV viral load in breast cancers with prior cervical neoplasia. The identification of koilocytes by light microscopy is subjective. It is difficult to distinguish between true koilocytes, fixation artifacts, and apoptopic epithelial cells. However, koilocytes were identified in approximately one-third of breast cancer specimens, which were positive for HPV by *in situ* PCR, which adds validity to the histological assessments.

The observation that HPV E7 protein expression may be absent in HPV sequence positive breast cancer specimens is of interest. A possible explanation is that HPV infections may have an early role in breast oncogenesis. This view is supported by the recent findings by Ohba et al. (18) that HPV infections appear to enhance the biological activity of APOBEC3B genes, which are known to lead an increased risk of breast cancer (19).

Although it is accepted by research workers in this field that HPVs are present in many breast tumors, there is concern that the viral load is so low that HPVs may not be oncogenic in breast cancer (20). However, similarly, low HPV viral loads have been observed in some cervical cancers, which indicate that low viral loads of HPVs can be oncogenic (16). In addition to the role of HPVs in the enhancement of the biological activity of APOBEC genes early in breast cancer oncogenesis adds to the validity of this concept. Epstein–Barr virus has been identified in 68% of Australian invasive breast cancers and may also enhance the oncogenicity of HPV despite this low viral load (21).

The expression of HPV E7 proteins and HPV-associated koilocytes in these sets of specimens are an indication that HPVs in these breast specimens are biologically active and capable of being oncogenic. Accordingly, despite their extremely low viral load, HPVs appear to be biologically active and have oncogenic influences in some of the breast cancers in this study.

Of particular interest is the very young age of many of these patients. Approximately 39% of the patients with prior cervical neoplasia were 45 years of age or below at age of breast cancer diagnosis. Breast cancer in young women is often aggressive and appears to have distinct biological characteristics (22). These include a core set of abnormally regulated microRNAs that differ from microRNAs in breast cancer in older women (22). The young age of these patients parallels the high prevalence of high-risk HPV infections among young Australian women (8). For Australian women aged 15–40 years, the prevalence of cervical HPV infections prior to HPV vaccination programs was 41% and over 50% for women under the age of 30 years (8). The implication is that young age breast cancer may be associated with high-risk HPV infections.

There has been a small but significant increase in the incidence among young (below age 40 years) US women of breast cancers with metastases during the past three decades (23). The 5-year mortality rate among these young women is approximately 70%. This increase in serious young age breast cancers parallels the increase in HPV genital infections in young US women. Again the implication is that high-risk HPV infections may be the reason for the increase in breast cancer in young women.

These observations are in accord with the well-known bimodal peak frequencies of breast cancer at different ages of diagnosis (23). The first peak tends to arise early in life at around age 50 years and generally behaves aggressively. The second peak occurs later in life at around 70 years and usually follows a more indolent course.

Also of interest is the 3.7-year gap between age of cervical and breast cancer diagnosis among women aged below 50 years as compared with 9.0 years for women 50 years and over at age of breast cancer diagnosis. This is compatible with the greater influence of sex hormones among younger women on both breast cancer and HPV viral replication (24). The HPV genome contains sex hormone receptors and replication is enhanced in the presence of sex hormones (25). The HPV18 regulatory region contains one functional GRE sequence that interacts with the glucocorticoid receptor and confers hormonal activation to the HPV18 P105 promoter (25). This is also compatible with the trend to higher grades of breast cancer among younger women with high hormone levels.

The mode of transmission of HPV to the breast is not known. However, it is possible that HPVs are transmitted to the genital tract during sexual activities and later transmitted by white blood cells throughout the body, including the breasts. This hypothesis is based on the young age of women with HPV positive breast cancer and who are sexually active and have a high incidence of HPV cervical infections plus the repeated identification of high-risk HPVs in white blood cells (26).

Other viruses, including mouse mammary tumor virus, Epstein–Barr virus, and bovine leukemia virus, may each have a role in breast cancer including collaborative roles with HPVs (21). Therefore, it is difficult to determine the proportion of breast cancers that may be caused by high-risk HPVs. However, as described above, this proportion is probably very small.

## Conclusion

The findings in this study indicate that high risk for cancer HPVs can be associated with cervical neoplasia and subsequent young age breast cancer. However, these associations are unusual and are a very small proportion of breast cancers. These outcomes confirm and extend the observations of two similar previous studies and offer one explanation for the increased prevalence of serious invasive breast cancer among young women.

The availability and widespread use of vaccines which can effectively control high-risk HPV infections may result in a decrease of HPV-associated breast cancer and, thereby, provide evidence whether HPVs have a causal role in breast cancer.

## Ethics Statement

This project has formal ethics approval by the University of New South Wales Human Research Ethics Committee – number HREC HC11421. Participants gave written informed consent to participate in this study. Ethics approvals for the follow-up of women who donated normal breast tissues was given by the New South Wales Population and Health Services Research Ethics Committee – number 2009/12/203. This Ethics committee waived the need for consent. The reasons for waiving consent were (i) the specimens were archival having been collected in 1999, 2000, and 2001, (ii) all specimens were “de-identified” to the research group, and (iii) retrospective approaches to donors, all of whom had cosmetic surgery, may have caused unnecessary anxiety.

## Conflict of Interest Statement

The authors declare that the research was conducted in the absence of any commercial or financial relationships that could be construed as a potential conflict of interest.
